# Preference and willingness to pay for traditional medicine services in rural ethnic minority community in Vietnam

**DOI:** 10.1186/s12906-016-1010-7

**Published:** 2016-02-03

**Authors:** Bach Xuan Tran, Ngan Kim Nguyen, Lan Phuong Nguyen, Cuong Tat Nguyen, Vuong Minh Nong, Long Hoang Nguyen

**Affiliations:** 1 Institute for Preventive Medicine and Public Health, Hanoi Medical University, Hanoi, Vietnam; 2 Johns Hopkins Bloomberg School of Public Health, Johns Hopkins University, Baltimore, MD USA; 3 T.H.Chan School of Public Health, Harvard University, Cambridge, USA; 4 Department of Epidemiology, Hanoi School of Public Health, Hanoi, Vietnam; 5 Institute for Global Health Innovations, Duy Tan University, Da Nang, Vietnam; 6 School of Medicine and Pharmacy, Vietnam National University, Hanoi, Vietnam

## Abstract

**Background:**

Traditional medicine (TM) still plays an important role in a number of health care systems around the world, especially across Asian and African countries. In Vietnam, however, little is known about preference for traditional medicine use. This study assessed the prevalence of use, preference, satisfaction, and willingness to pay for TM services amongst rural ethnic minority community.

**Methods:**

A cross-sectional survey in three provinces in the North and South of Vietnam.

**Results:**

The results showed a high level of satisfaction with TM services, with more than 90 % of respondents reporting improved health status given the use of TM. Indicators for preference of TM over modern medicine are a longer distance to health station; being in an ethnic minority; being female; and having had higher service satisfaction. Although we did not have a comparison group, the high level of satisfaction with TM services is likely the result of a project targeting community health workers and the public regarding TM education and access promotion. Indeed, the community health workers are credited with relaying the information about TM more than any other sources. This suggests the importance of community health workers and community health centers in the promotion of TM use.

**Conclusions:**

Ethnic minority people prefer the use of traditional medicine services that supports the expansion of national programs and promotion of traditional medications.

## Background

Traditional medicine (TM) is defined as “the sum total of the knowledge, skills, and practices based on the theories, beliefs, and experiences indigenous to different cultures, whether explicable or not, used in the maintenance of health as well as in the prevention, diagnosis, improvement or treatment of physical and mental illness” [1]. TM’s use is widespread not only in developing countries of Africa, Asia and Latin America but also in developed countries. The prevalence of TM use by general populations was higher in East Asian countries (South Korea: 45.8–69 %, China: 90 % or Lao PDR: 77 %) [2–4] in comparison with developed countries with around 50 % in Australia, 42 % in USA and 49 % in France [5]. Higher TM use was found to be associated with female [6, 7], higher level of education [6, 8–12], higher age [7], and poorer health status [7, 13].

The preference for TM and its reasons were diverse across regions. In both developed and developing world, for example, Uganda, Ethiopia, and the United States, there was about 45 to 70 % people more likely to choose traditional medical care than western medicine [10, 11, 14]. Among those given preference for TM, they highly appreciated its affordability, accessibility, and acceptability [14]. However, the choice of patients and treatment outcomes using TM differed significantly between patient groups with different diseases [15]. A review of TM in European countries showed that the majority of patients who chose to use TM had experienced ineffective and dissatisfied conventional treatments using western medicine [16]. In addition, the use of TM was associated with awareness of patients, availability of services and coverage of communication campaigns on TM [13, 17, 18].

Vietnam is located on the eastern rim of the Indochina peninsula in the South East Asian tropical monsoon zone. The country has a long history of traditional medicine which was formed throughout thousands of years of national construction and defence [19]. The government of Vietnam regards TM as one of national cultural inheritances needed to be preserved and developed. In 2010, Government issued the Master Action Plan on development of TM up to 2020 to boost the TM development and usage [20]. However, people in mountainous and difficult areas still had limited access to TM because of various factors including their limited knowledge and awareness on TM; sparse population distribution on difficult-to-reach terrains; long distance to the health centre; and unsatisfactory service (Tran Xuan Bach, Nong Minh Vuong, Cuong NT: Post-implementation evaluation of the model using traditional medicine kits in selected communes in Vietnam, Unpublished work).

Although there were several studies that examined TM use in the world, the limited scientific evidence regarding awareness and preference of TM make it important to conduct research specific in Vietnam. This paper aims to assess preference and awareness of traditional medicine use as well as the satisfaction for TM services among people in mountainous and difficult areas. The results can also serve to inform future research direction into traditional medicine in Vietnam [21].

## Methods

### Study setting, study subjects and sample size

This cross-sectional study was conducted in three provinces including Hoa Binh, Quang Ninh and Dong Nai. Two communes in resource-limited settings of each province were purposively selected for the survey. These communes are all in mountainous or remote areas and have a distance of 10 to 40 km away from a district health centre. We randomly selected 5 villages in each commune from which we conveniently select 10 households, making a total of 50 households per commune.

### Measurements and data collection

We conducted face-to-face interviews using a structured questionnaire to collect information about socioeconomic, awareness, TM services utilization, knowledge, attitude and practice of using TM among respondents. We also asked the preference and willingness to pay for TM kit, which was assumed to include from ten to twenty varieties of Vietnamese TM (for some common diseases such as cold, fever, diarrhea, muscle pain, etc), several small medical equipment such as thermometer, cotton gauze, bandages, and a guidebook.

Perceived health improvement by traditional medicine, self-evaluated knowledge and competency on traditional medicine use were measured using a five-level scale ranging from 1 to 5. Participant’s satisfactions with services quality included 10 questions on which respondents rated on a 10-point scale, namely: 1) overall satisfaction with general services at commune health station, 2) overall satisfaction with TM services, 3) access to information, 4) consultation, explanation, and guidance, 5) convenient in using TM, 6) convenience in combining traditional and western medicine, 7) inter-professional collaborations at health stations, 8) competence - and 9) responsiveness of TM doctors and health workers 10) availability of demanding TM services. Well-trained interviewers, who are master students at Hanoi Medical University, with supports by village health workers, visited households and invited family head or any other people at home to participate in the survey.

### Data analysis

We used Stata 12.0 to produce both descriptive and analytical statistics. A p-value of less than 0.05 was considered statistical significance. We used censored regression model to find the significant predictors of preference for and health improvement by TM services.

### Ethical consideration

The Scientific Research and Ethics Committee of National Occupational and Environmental Health approved the protocol. We obtained written informed consent from all participants after clearly introducing the survey. Respondents could refuse to participate or withdraw from the interview at any time, and this did not affect their continuation of services. Confidentiality was assured using codes of their information, and secured storage was prepared for both paper questionnaires and electronic data set.

## Results

Table 1 presents characteristics of respondents participated in the study. The mean age of all participants was 46.5 (SD = 13.0); 64.7 % were female; 40.7 % had secondary school education and above. There were 56.4 % respondents who reported a distance over 2 km to the commune health station.

**Table 1:** Characteristics of respondents in three provinces

	HoaBinh		QuangNinh		Dong Nai		Total		*p*
	Mean	SD	Mean	SD	Mean	SD	Mean	SD	
Mean age	42.6	13.6	47.2	13.9	50.7	11.9	46.5	13.0	<0.001
	N	%	N	%	N	%	N	%	
Female	54	54.0	70	70.0	70	70.0	194	64.7	<0.05
Age groups									<0.001
20–30	24	24.0	12	12.0	6	6.0	42	14.0	
31–40	17	17.0	22	22.0	16	16.0	55	18.3	
41–50	34	34.0	29	29.0	21	21.0	84	28.0	
41–60	17	17.0	19	19.0	37	37.0	73	24.3	
> 60	8	8.0	18	18.0	20	20.0	46	15.3	
Educational attainment									
Primary school	38	38.0	36	36.0	62	62.0	136	45.3	<0.001
Secondary school	45	45.0	28	28.0	16	16.0	89	29.7	
High school and above	11	11.0	9	9.0	13	13.0	33	11.0	
Others	6	6.0	27	27.0	9	9.0	42	14.0	
Number of family members									
1 to 2	6	6.0	24	24.0	25	25.0	55	18.3	<0.001
3 to 4	44	44.0	44	44.0	48	48.0	136	45.3	
> = 5	59	59.0	32	32.0	27	27.0	118	39.3	
Average income (VND)								0.0	
< 20 millions	41	41.0	51	51.0	12	12.0	104	34.7	<0.001
20–60 millions	57	57.0	40	40.0	64	64.0	161	53.7	
> 60 millions	2	2.0	9	9.0	24	24.0	35	11.7	
Distance to health community station									
< 1 km	16	16.0	28	28.0	3	3.0	47	15.6	<0.001
1–2 km	34	34.0	37	37.0	13	13.0	84	28.0	
> 2 km	50	50.0	35	35.0	84	84.0	169	56.4	

Table 2 presents services utilization characteristics, participants’ self-evaluation of knowledge and skills on TM, and perceived health improvements by gender. We found that the major source of information about traditional medicine included community health workers (90.3 %), followed by relatives, neighbours or friends (26.3 %). The media (TV, newspaper) and traditional healers (including pharmacies that specialize in medical herbs) play less prominent roles in dispersing information on TM, accounting for 9.7 and 8.7 % of the information gained respectively. There were 61.4 % men and 55.3 % women who reported that they had levels of very good or good of knowledge and skills on the use of traditional medicine for their health care. About 91.6 % of respondents reported that they perceived an improvement in their health status given the use of traditional medicine. Almost two thirds of participants (66.3 %) claimed that with TM, they were “much better, but not completely cured”. Only 11.3 % claimed that they were completely cured; while others reported feeling “a little bit better” (14 %), “unknown” (6 %), or “not better but not worse” (2.3 %).

**Table 2:** Services utilization and self-evaluated knowledge and skills on traditional medicine by sex

	Male		Female		Total		*p*
Source of Traditional medicine information	N	%	N	%	N	%	
— Community health workers	99	93.4	172	88.7	271	90.3	0.18
— Traditional healers	8	7.5	18	9.3	26	8.7	0.61
— TV/paper	15	14.2	14	7.2	29	9.7	0.89
— Relatives, neighbors or friends	25	23.6	54	27.8	79	26.3	0.42
Traditional Medicine Package Use	Mean	SD	Mean	SD	Mean	SD	
— Frequency of use (times/year)	6.74	5.3	5.73	3.5	6.1	4.2	0.03
— Frequency of refiling the traditional medicine	3.03	2.6	2.81	2.2	2.89	2.3	0.22
Self-evaluated knowledge on traditional medicine use	N	%	N	%	N	%	
— Very good	15	14.2	13	6.7	28	9.3	0.01
— Good	50	47.2	94	48.5	144	48.0	
— Moderate	27	25.5	71	36.6	98	32.7	
— Poor	11	10.4	16	8.3	27	9.0	
— Very poor	3	2.8			3	1.0	
Self-evaluated skills on traditional medicine use	N	%	N	%	N	%	0.14
— Very Competent	16	15.1	16	8.3	32	10.7	
— Fairly Competent	48	45.3	93	47.9	141	47.0	
— Moderately Competent	31	29.3	63	32.5	94	31.3	
— Little competent	8	7.6	21	10.8	29	9.7	
— Not at all Competent	3	2.8	1	0.5	4	1.3	
Perceived health improvement by traditional medical use	N	%	N	%	N	%	
— Completely cured	15	14.2	19	9.8	34	11.3	<0.001
— Much better but not completely cured	67	63.2	132	68.0	199	66.3	
— A little bit better	20	18.9	22	11.3	42	14.0	
— Not better but not worse	0	0.0	7	3.6	7	2.3	
— Unknown	4	3.8	14	7.2	18	6.0	

In Table 3, we found that respondents were highly satisfied with all criteria of quality of the traditional medicine services, indicated by scores over 8 in a 10-point rating scale. Men reported slightly higher levels of satisfaction on traditional medicine services than women across all ten dimensions of the services quality measure, and are also willing to pay more. On average, men would pay for about VND 589,000 (~ USD 28) and women would pay VND approximately 120,000 (~USD 6) for one-year use of the traditional medicine box with multiple refills. Two thirds of respondents have a preference for traditional medical care over modern medicine.

**Table 3:** Respondents’ satisfaction and willingness to pay for traditional medicine services by sex

	Male		Female		Total		*p*
Satisfaction on TM services	Mean	SD	Mean	SD	Mean	SD	
— Overall satisfaction with general services quality at commune health station	8.83	1.10	8.75	1.15	8.78	1.13	0.28
— Overall satisfaction with traditional medical services quality	8.36	1.33	8.04	1.54	8.15	1.47	0.03
— Access to informations about TM	8.88	1.12	8.64	1.24	8.73	1.20	0.05
— Consultation, explanation, and guidance on TM use by health workers	8.79	1.19	8.68	1.23	8.72	1.22	0.23
— Convenience in using traditional medicine package	9.12	1.03	9.10	1.02	9.11	1.02	0.44
— Convenience in combining traditional and western medicine	8.76	1.13	8.49	1.28	8.59	1.24	0.03
— Inter-professional collaborations at health stations	8.66	1.09	8.32	1.36	8.44	1.28	0.01
— Competence of TM doctors and health workers	8.48	1.24	8.10	1.39	8.23	1.35	<0.001
— Responsiveness of TM doctors and health workers	8.76	1.11	8.67	1.35	8.71	1.27	0.28
— Availability of demanding TM services	8.32	1.50	8.30	3.40	8.30	2.87	0.47
Willingness to pay for TM package	N	%	N	%	N	%	
— Willing to pay	100	94.3	171	88.1	271	90.3	0.08
— Not willing to pay	6	5.7	23	11.9	29	9.7	
	Mean	SD	Mean	SD	Mean	SD	
Mean price (thousand VND)	589.13	3146.86	119.94	115.06	289.88	1902.98	0.03
	N	%	N	%	N	%	
<100 thousand VND	6	5.7	75	38.7	81	27.0	<0.001
100–300 thousand VND	65	61.3	70	36.1	135	45.0	
>300 thousand VND	35	33.0	49	25.2	84	28.0	
	N	%	N	%	N	%	
Preference for traditional medicine over modern medicine	64	60.4	133	68.9	197	65.9	0.14

In Table 4, we identified factors associated with the participants’ preference for traditional over modern medicine and perceived health improvement given the use of traditional medicine. We found that respondents who were Muong people (vs. Kinh people), female, more competent in traditional medicine use, or who had longer distance to commune health station reported higher preference traditional medicine than their counterparts. In addition, services satisfaction was significantly related to the preference for traditional medicine. Particularly, respondents with higher satisfaction with traditional medical care services, who experienced convenience when using traditional medicine box, or who thought that their traditional medicine doctors were responsive, were more likely to choose traditional medicine rather than western medicine. Adjusting for other covariates, we found that perceived health improvement was a strong predictor of respondents’ preference for traditional medicine. In censored regression model, we found that health improvement given traditional medicine use was positively associated with higher education, knowledge on traditional medicine and its ease to use; whereas it was negatively associated with longer distance to commune health stations.

**Table 4:** Factors associated with preference for and health improvement by traditional medical services

	Prefer traditional over modern medicine			Perceived health improvement by traditional medicine		
	OR	95 % CI		Coef	95 % CI	
% Spending on health care						
Lowest (ref)						
Moderate				0.29	−0.02	0.59
High				0.25	−0.02	0.51
Ethnics: Muong vs. Kinh	2.22*	1.05	4.72			
Sex: female vs. male	2.27*	1.09	4.72			
Education: Secondary school and above vs. below				0.29*	0.06	0.52
Distance to health station						
< 1 km (ref)						
1–2 km				−0.20	−0.49	0.10
> 2 km	2.17*	1.10	4.29	−0.30*	−0.58	−0.01
Health problems: Yes vs. No				−0.23	−0.47	0.01
Knowledge on TM use	0.50	0.21	1.22	0.24*	0.10	0.37
Competency on TM use	2.44*	0.99	6.01			
Perceived health improvement given TM use	1.65*	1.02	2.75			
Services Satisfaction						
— Overall satisfaction with general services quality at commune health station	0.68	0.41	1.12			
— Overall satisfaction with traditional medical services quality	1.72*	1.10	2.67			
— Consultation, explanation, and guidance on TM use by health workers	0.60	0.34	1.07			
— Convenience in using traditional medicine package	1.81*	0.99	3.31	0.16*	0.02	0.30
— Convenience in combining traditional and western medicine	0.70	0.44	1.14			
— Inter-professional collaborations at health stations				−0.15	−0.32	0.02
— Competence of TM doctors and health workers				0.11	−0.03	0.26
— Responsiveness of TM doctors and health workers	1.62*	1.01	2.68			
Constant				1.98	0.92	3.04

* *p* < 0.05

## Discussion

This study offers some insights into the awareness and preference of traditional medicine services in three rural, mountainous provinces of Vietnam: Hoa Binh, Quang Ninh, and Dong Nai. Except for Phu Ly, the communes selected in these provinces share the same characteristic of having at least a third of their population living in poverty. In Hoa Binh, ethnic minority groups make up more than 70 % of total population, in which 63.4 % is Muong, followed by Thai (3.9 %), Tay (2.7 %), Dao, and H’Mong. Selected communes are located at a distance of 10 to 40 km away from the district health center, which hints at difficulties in getting access to health care and subsequent low uptake of service [22].

### Knowledge and skills in traditional medicines

Results show that community health care workers play an integral part in imparting knowledge in traditional medicine, and thus may take credits for their high self-evaluated knowledge and skills in traditional medicines. Friends, family and neighbors are also an important source for knowledge, possibly because participants live in small, rural communities, where social ties are deeper and words of mouth have a major influence in which services people choose to use [23].

Both the media and traditional healers do not seem to be major factors in people’s knowledge of traditional medicines. In this study, “traditional healers” include pharmacies that specialize in medicinal herbs, and while these facilities may have more influence over information on TM in other countries in Asia, they seem to have the least impact in distributing this information in the study.

These results likely indicate the early outcomes of the Traditional Medicines project, which aimed at building capacity for community health care workers and promote knowledge among the community on traditional medicines. A vast scientific literature has regarded community health workers as an integral part of the health care system [24–26], especially in low and middle-income countries. They serve as health extenders [25], natural healers who build rapport, trust, and understanding between the patients and the health care system [24], and important members of the health care workforce who enhance the quality of life for people in poor, marginalized, and underserved area [25]. The results of this study corroborate the literature claim of community health workers’ role in a subdomain of the health care system: traditional medicine. When it comes to health promotion, the rural, mountainous setting of the three provinces in the study calls for community health workers’ mobility, accessibility and local knowledge, thus it is crucial to tap on this resource. Community health care workers when described in this context are usually based in community health centers, which reiterate the importance of these facilities in providing care to populations living in the rural, mountainous areas.

### Willingness to pay

The participants’ high willingness to pay carries more significance if we consider the percentage of participants in this study who live in poverty (at least 30 %, except for Phu Ly). There was no data related to social or health insurance coverage for these participants, thus it is not clear whether they will be reimbursed for these purchases. It suggests that they think the traditional medicine box is good value for money for their households. Logistics may serve as another reason for the willingness to pay. Literature has indicated that long distance to health facilities acts as a clear-cut barrier to people’s service utilization [22]. Therefore, besides the perceived good quality of traditional medicine services, the lack of frequent access to health facilities may serve as yet another motivator for people to obtain a traditional medicine box, since it would potentially spare them the trouble of going back to the district health center every time they have an ailment.

The fact that men were willing to pay more for this medicine box may bear several possible explanations: men’s financial authority in the household, especially in the countryside of Vietnam, where patriarchal ideologies still pertain; women’s tendency to be more economical as the role of the family “caretaker”. It would be interesting to explore this discrepancy in willingness to pay and how gender plays a role in men’s and women’s behaviors in spending for traditional medicine.

### Preference

We observe that the Muong people, an ethnic minority, are twice more likely to prefer traditional over modern medicine. Literature has shown the importance of traditional spiritual beliefs among ethnic minorities in daily life in general and health care in particular [27].

Various studies in the South East Asia and East Asia region have also indicated high prevalence of traditional medicine use among ethnic minorities [28, 29]. However, we lack data that indicates whether ethnicity is a clear predictor of the preference of traditional over modern medicine in the region. Logistics is again a deciding factor in people’s choice, since those who live farther away (>2 km) from a commune health center tend to choose traditional medicine services (OR = 2.17; 95 % CI 1.10–4.29), where they can subsequently take up a medicine box for use at home.

Perceived health improvement is imperative and is predicted by participants’ knowledge of traditional medicine and its simplicity to use. These ‘convenience’ and ‘simplicity’ characteristics suggest that traditional medicine are most likely used for self-management of existing medical conditions and health maintenance. Studies on traditional medicines in Uganda and Malaysia [28] both indicate the same result. In a study by Jung Hwang et al. on the utilization of traditional medicine among ethnic minorities in South Korea [29], it is found that traditional medicine is most frequently employed in treating cold, fatigue, stomach pain, and joint pain. In Vietnam, self-management of diseases is also recorded and we observed that TM was used to treat back pains, headache, cold, flu, joint pain, inflammation and digestive disorders, which were the common diseases in population.

It is worthwhile to note that this study only looks at the preference of traditional medicine over modern medicine, and not looking at them as complementary. In the same study by Hwang et al., it was found that 55 % of the participants felt that traditional medicine was effective or that the combination of conventional (modern) and traditional medicine was more effective than conventional medicine alone. The same results were found in a study on traditional medicine use in Mongolia, with 38 % combined use [30]. It would be interesting to look more into this behavior of respondents in future studies.

### Policy implications

This study shows the importance of community health centers in general and community health care workers in particular in dispersing information and awareness on traditional medicine to people in rural and mountainous areas. The community health centers can serve as hubs where people can get reliable information on traditional medicine purchase the medicine box for home use, and consult physicians or pharmacists about their usage of traditional medicine. This suggests the need to invest in building and maintaining community health care centers, as well as to support community health care workers financially (through salary and allowance) and professionally (through training and technical assistance) if we want to promote traditional medicine in these rural areas. Community network is also a crucial channel to relay information about traditional medicine health services, as shown in the study. A community awareness component therefore should be put in place once services have been established to ensure people get another sources of information on traditional medicine [31].

Additionally, while people indicated the high effectiveness of TM, health workers should provide more knowledge and skills about how to use TM appropriately. Some evidences suggested adverse effects of TM, especially in treating infectious diseases due to the nature of TM and the infections [32, 33]. Therefore, the instruction of health workers had a central role to improve the impact of TM on health status of population.

As observed in the study, the convenience feature of the traditional medicine box was highly appreciated by participants, and is worth noting to implement further interventions of TM kit provision for this population. This study also suggests further research on the willingness to pay for traditional medicine and the role of insurance in defraying this cost.

### Strength and weakness of study

This is one of the first studies to look at traditional medicine use in rural, mountainous parts of Vietnam, and also the first to explore the awareness and preference of traditional over modern medicine. Although the results of this study strongly suggest the initial impact of the traditional medicine promotion project on people’s high awareness of preference regarding TM, we cannot draw causal inference due to the lack of a comparison group. The sampling method employed in this study also may lead to lack of representation from disenfranchised groups in rural areas.

## Conclusion

In conclusion, we found a strong preference and willingness of respondents to use and to pay for TM in rural areas of Vietnam. Strengthening TM service quality and availability, improving the convenience in TM use and increasing awareness and skills of local people are necessary to promote TM in primary health care.
